# Use of Topical Nitroglycerin in the Treatment of Acute Limb Ischemia: Four-Year Experience in a Mixed Neonatal and Pediatric Intensive Care Unit

**DOI:** 10.34763/jmotherandchild.20263001.d-25-00039

**Published:** 2026-03-06

**Authors:** Ana Rita Jesus, Ana Rita Fradique, Catarina Teixeira, Leonor Carvalho, Teresa Dionísio

**Affiliations:** Pediatric Intensive Care Unit, Department of Paediatrics, Unidade Local de Saúde de Coimbra, Coimbra, Portugal

**Keywords:** Acute Limb Ischemia, Nitroglycerin, Pediatric Intensive Care Unit, Neonatal Intensive Care Unit

## Abstract

**Background:**

Pediatric Acute Limb Ischemia (ALI) is a rare condition, sometimes encountered in the Intensive Care Unit (ICU). Optimal treatment remains to be established. Topical nitroglycerin has been suggested as a vasodilator agent, used to improve limb reperfusion.

**Materials and Methods:**

We performed a retrospective descriptive study, with data from a PICU of a tertiary hospital, from 2021 to 2024.

**Results:**

Seven children presented with ALI treated with topical nitroglycerin, with a median age of 244 days. Wrongful arterial catheterization was established as the cause of ALI in four out of seven children. Six out of seven children showed at least partial recovery. Complete recovery was achieved in three children. Three children underwent partial or total amputation. No adverse side effects were recorded.

**Conclusion:**

Topical nitroglycerin appears to be a beneficial and safe intervention in children with ALI.

## Introduction

Acute Limb Ischemia (ALI) is a rare condition that initially presents as pallor of the affected limb with no palpable pulses. It subsequently progresses to discoloration or greyish hue, and eventually to irreversible necrosis [1]. These clinical signs form the basis for the diagnosis of ALI [2], although Doppler ultrasonography may also play a role in identifying thrombosis [2]. Prompt diagnosis is crucial, as ALI may result in limb amputation or deformation with loss of function [3], and in some cases may even pose a threat to the patient life [2].

Although epidemiological studies are scarce, there is general consensus that the most common cause of ALI is iatrogenic, most often due to misplaced arterial catheterization [2]. This procedure is frequently performed in critically ill children in the Intensive Care Unit (ICU) setting [1]. Obstruction of blood flow by thrombi, emboli, or vasospasm, often caused by vasoactive drugs or direct damage to the arterial wall during catheterization, as well as extravasation of intravenous drugs, may all lead to ischemic lesions [3, 4]. Other possible causes of ALI are prolonged hypoperfusion due to shock and purpura fulminans [5]. Conditions that predispose to a prothrombotic state, such as sepsis and dehydration [6], may increase the risk of ALI.

Prematurity and low birth weight are thought to increase the risk of poorer ischemic outcomes, due to the overall fragility of the patient, the smaller caliber of their blood vessels, and the overall paucity of evidence for effective treatments and procedures [7].

Treatment for ALI, particularly in the pediatric setting, lacks consensus, with most data originating from case reports, as this is fortunately a rare condition [4]. Optimal management depends on anatomical location, extension and mechanism of ischemia [1]. Furthermore, these patients often present with other conditions and risk factors, such as hemodynamic instability or coagulopathy, that may limit therapeutic options [8]. While there is no standardized approach (8), both pharmacologic and non-pharmacologic interventions have been proposed in literature.

Non-pharmacologic interventions include the removal of the arterial catheter, limb elevation and soft massage, and the application of warm compresses to the contralateral limb (7, 9). However, the efficacy of these interventions when used in isolation to treat ALI seems insufficient (7). Consequently, pharmacological agents have been proposed. The first documented description of nitroglycerin (NG) utilization for the treatment of ischemia in newborns is from 1988 (9). Since then, it has been used off-label in ICUs worldwide, despite a lack of global consensus on dosage and preferred formulation. Side effects, such as methemoglobinemia and hypotension, are rare, making topical NG a safe alternative to use alone or with other agents, most commonly unfractionated heparin or low molecular weight heparin.

Treatment success of ALI in the pediatric setting, appears to be multifactorial. To date, no international consensus exists regarding the optimization of care provided to affected children, as further data are required to build such recommendations. Furthermore, most studies report cases of iatrogenic ALI, leaving out ischemia attributable to hypovolemic shock. This study reports the management of ALI with topical NG in a Portuguese mixed Neonatal and Pediatric Intensive Care Unit (PICU) of a tertiary pediatric hospital.

## Materials and Methods

A retrospective descriptive study was designed, with data collected from 2021 to 2024. All cases of ALI from non-traumatic causes (i.e., ischemia not caused by external trauma but typically related to catheter-associated obstruction, vasospasm, or prolonged hypoperfusion) were retrospectively identified from a local database, in accordance with our hospital’s archival research policy. Data regarding demographic characteristics, comorbidities, ICU stay, and treatment provided were collected. Confidentiality was assured by attributing a non-identifiable number to each child. Written informed parental consent was obtained for the publication of the images included in this paper.

Statistical analysis was performed using Microsoft Excel (Microsoft Corporation, Redmond, WA, USA). Median is presented for continuous variables, with the minimum and maximum values in brackets [minimum-maximum].

## Results

Between 2021 to 2024, our PICU registered a total of 1,596 admissions. During this period, ten children were diagnosed with non-traumatic ALI, for an incidence rate of 2.5 cases per year, or 1 per 160 children admitted. Three children were not treated with topical nitroglycerin. Amongst those, we found two children that presented with cardiogenic shock and multiorgan failure. Both developed iatrogenic ALI after catheterization but unfortunately died before initiation of topical treatment. The third child presented with severe septic shock with multiorgan failure and infectious gangrene of both lower limbs, requiring lifesaving early amputation, which rendered NG unnecessary.

A total of seven cases of ALI were treated with NG over a four-year period. The demographic and comorbidity data are presented in Table 1. Most children were male (four children), reflecting the predominantly male percentage of children admitted in the ICU (55.7%). Median age at PICU admission was 244 days [22 days – 15,5 years]. Four children were admitted after surgery for postoperative care. All children were ventilated, although ventilation times varied significantly, with a median of 25 days [1 – 53]. All but one needed inotropic support, with a median of 15 days [4 – 43]. Acute Kidney injury was present in six out of seven children, with half of those requiring renal replacement therapy. Six out of seven children were treated for sepsis. The median length of ICU stay was 51.5 days [3 – 109].

**Table 1.** PICU patients treated with nitroglycerin – demographic and comorbidity data.

**No.**	**Year of admission**	**Sex**	**Age, days**	**Reason for PICU admission**	**IMV, days**	**Inotropic support, days**	**Renal replacement therapy**	**Sepsis**	**Length of stay, days**
1.	2022	M	315 (10.5 months)	Postoperative care (cardiac surgery)	17	7	NN	+	†
2.	2022	M	505 (16.8 months)	Postoperative care (cardiac surgery)	33	43	HDF + peritoneal dialysis	+	41
3.	2023	F	73 (2.4 months)	Postoperative care (cardiac surgery)	28	25	NN	+	62
4.	2023	F	5692 (15,5years)	Acute liver failure	*	4	HDF	+	109
5.	2024	M	106 (3.5 months)	Postoperative care (craniosynostosis surgery)	1	-	No AKI	-	3
6.	2024	M	22 (0.7 months)	Congenital heart disease with cardiogenic shock	53	23	HDF	+	63
7.	2024	F	244 (8.1 months)	Bronchiolitis with ARDS	18	6	NN	+	31

AKI = Acute Kidney Injury; ARDS = Acute Respiratory Distress Syndrome; F = female sex; HDF = hemodiafiltration; IMV = Invasive Mechanical Ventilation; M = male sex; NN = none needed; PICU = Pediatric Intensive Care Unit;

* = a tracheostomy was performed, after which the child was discharged home under domiciliary mechanical ventilation;

† = the child died in day nine of admission.

Data regarding ALI etiology, treatment and outcome are presented in Table 2. The diagnosis of ALI was based on clinical signs, with additional support from Doppler sonography evaluation, performed in all patients. ALI was established after wrongful arterial catheterization in four children. All inserted catheters were removed after ALI diagnosis. Prolonged hypoperfusion accounted for the remaining three ALI cases.

**Table 2.** PICU patients treated with nitroglycerin – ALI location, management and outcome.

**No.**	**ALI etiology**	**ALI location**	**NG administration**	**NG started**	**Treatment duration, days**	**ACT**	**ACT, days**	**ABX**	**Maximum MetHb value, %**	**Outcome**
1.	PH	Extremities of all four limbs (hands and feet)	NG patches of 5 mg every 48 hours	D6	†	LMWH	†	+	0.4	Stopped ischemia progression (child died of non-ALI related complications)
2.	AC	Right hand fingers	NG ointment 4 mg/g every 24 hours	D0	12	NFH → LMWH	28	+	1.2	Stopped ischemia progression. Partial recovery, partial amputation of 4^th^ finger
3.	PH	Fingers of both feet	NG ointment 4 mg/g every 24 hours	D1	9	LMWH	28	+	1.6	Full recovery
4.	PH	Both legs, up to the knees	NG ointment 4 mg/g every 24 hours	D5	3	NFH → LMWH	89	+	1.9	No recovery, early amputation due to infectious gangrene
5.	AC	Right hand	NG ointment 4 mg/g every 12 hours	D1	2	LMWH	89	-	1.2	Full recovery
6.	AC	Left leg, up to the knee	NG ointment 4 mg/g every 12 hours	D0	17	LMWH	7	+	1.8	Full recovery
7.	AC	1^st^ to 4^th^ right hand fingers	NG ointment 4 mg/g every 12 hours	D5	20	NFH → LMWH	79	+	1.4	Stopped ischemia progression. Partial recovery, partial amputation of 1st finger

ABX = Antibiotics administered; AC = Arterial catheterization; ACT = Anticoagulation therapy; MetHb = Methemoglobin; NFH = Non-fractionated heparin; NG = Nitroglycerin; LMWH = Low molecular weight heparin; PH = Prolonged Hypoperfusion (as seen in hypotension due to shock);

* = time of initiation of nitroglycerin, counting from the day of ALI diagnosis;

† = the child died in day nine of admission.

All children were treated with topical NG. Only one child received a stick-on NG patch. This formulation was no longer available in our hospital since around mid-2022, leaving the other 6 children to be treated with rectal ointment 4mg/g, applied to all necrotic skin. Time to treatment initiation varied across the cohort of children, depending on clinical stability and treatment availability, with a median of 1 day [0 – 5]. Standard lesion dressing was performed after NG application. In cases where it was feasible, affected limbs were elevated. Full-dose anticoagulation therapy was initiated whenever clinically possible. Concerns about bleeding risk accounted for the differences in treatment duration amongst the cases, with a median of 53.5 days of anticoagulation [0 – 89]. Monitoring of anticoagulation therapy involved the assessment of activated partial thromboplastin time (aPTT) or anti-Factor Xa (anti-Xa) for non-fractionated heparin and low molecular weight heparin, respectively, with subsequent adjustment of dosage. Except for one child, all children received antibiotics due to other conditions. Pain management drugs were administered to all children throughout all treatment duration.

Adverse side effects were actively monitored. Blood pressure was measured hourly during the duration of treatment. No significant decrease in mean blood pressure after treatment initiation was recorded. Monitoring of blood methemoglobin levels was conducted daily, with a median value of 1.4% [0.4 – 1.9]. No cases of significant methemoglobin levels (> 10%) were identified.

**Figure 1.**
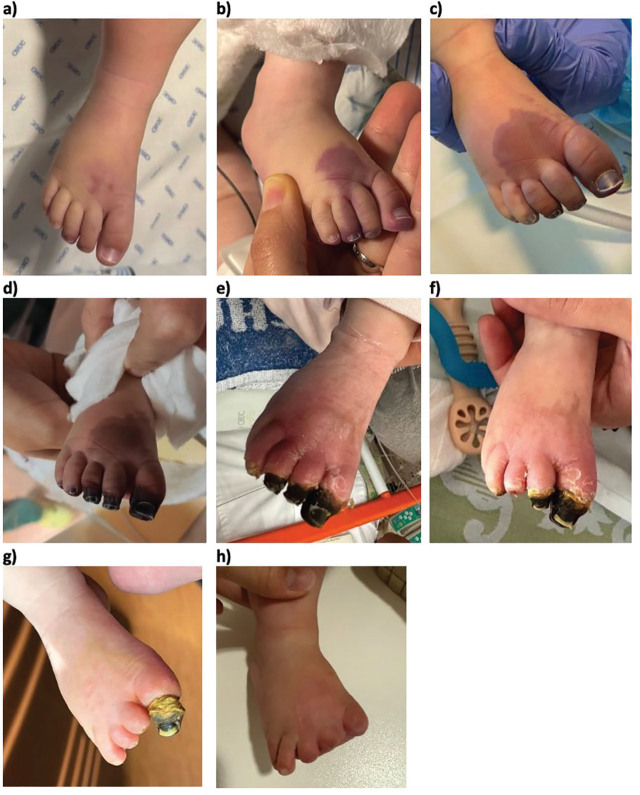
Progression of the ALI lesion in case 7. Sequential images illustrate the evolution of ischemia and its sequelae: a) day 1, first signs of ischemia; b) day 1, 12 hours after onset; c) day 3; d) day 5, initiation of treatment; e) day 35; f) day 49; g) day 73; h) day 252.

Full recovery was achieved in three out of seven children. Two children, cases number 2 and 7, showed partial improvement. Both cases underwent partial amputation after necrosis was set (one month and two and a half months after the initial ALI diagnosis, respectively), leaving the children with residual scarring without functional impairment. Figure 1 illustrates the progression of the ALI lesion in case 7. No conclusion could be made regarding treatment of the remaining two cases, as one suffered lifesaving early amputation due to infectious gangrene of both legs after only three days of nitroglycerin treatment, and the other died of cardiac arrest on the ninth day of nitroglycerin treatment. He had undergone complex cardiac surgery with immediate complications, and presented to postoperative care with hemodynamic instability, multiorgan failure and four limb ischemia. NG was started despite the poor prognosis, and even though the child died after only nine days of treatment, it did appear to have stopped the progression of ischemia.

## Discussion

### ALI incidence and physiology

Pediatric ALI is a rare but serious condition, often seen in the ICU setting. Reports on the incidence of ALI are scarce. A 2018 comparative study found an estimated incidence of 26 per 100,000 admissions (10). Another study (11) identified eight cases of catheter-related ALI over a three-year period in a tertiary hospital. A third study (12) reported an incidence of 2.4 cases per 1,000 neonates, in the neonatal ICU. In our study, we found an incidence rate of 2.5 cases of ALI per year, or 1 per 160 admissions to the ICU. We considered ALI from all causes except traumatic, as this has a different mechanism of injury and is usually managed by the Pediatric Orthopedics team. Different inclusion criteria may explain the discrepancies observed between studies regarding incidence, and further reports are needed to draw conclusions. Most studies report on ALI due to wrongful catheter insertion, indicating it as the cause for ischemia in up to 89% of cases (12, 13, 14). The insertion of catheters is a procedure frequently performed in the ICU setting, and may directly injure arteries, leading to thrombosis and vasospasm which in turn results in a reduction of blood flow to the affected limb, and subsequent tissue necrosis (11). In our study, we found wrongful catheterization to be the cause of ALI in four out of seven cases, which is consistent with literature available.

### Non-pharmacological treatment

Concrete data regarding optimal pediatric ALI treatment is still scarce, with some articles even advising conflicting recommendations (15, 16). The location and extent of ischemia, as well as patient status and comorbidities, have all been identified as factors that may influence treatment selection and its success rate (1). Non-pharmacological interventions have been proposed. Immediate catheter removal is recommended, as a prevention of further artery injury. It has been hypothesized that limb elevation and soft tissue massage may prevent the pooling of blood in the affected area, facilitating venous return, helping reperfusion (17). These measures, in conjunction with pain management, appear to be readily implementable and efficacious. Another non-pharmacological intervention frequently referenced in literature is contralateral limb warming. It has been hypothesized that this approach promotes reflex vasodilation in the affected limb, thereby enhancing reperfusion (9, 13). However, this is not a consensual measure, and is therefore rarely used (15). In our ICU, removal of all catheters in affected locations, pain management and limb elevation are offered to all ALI affected patients.

### Nitroglycerin treatment

Non-pharmacological measures alone appear to be ineffective in the treatment of ALI (13), and pharmacological interventions have been suggested, with topical NG being one of the most prominent. Topical NG is rapidly absorbed through the skin, where it acts by releasing nitric oxide. Nitric oxide activates guanylate cyclase, increasing the levels of cyclic guanosine monophosphate (cGMP), which in turn promotes smooth muscle relaxation and vasodilation, essential to limb reperfusion (3). As blood clots can also promote the release of vasoconstrictors and therefore exacerbate vasospasm, by acting as a promotor of vasodilation, NG can be useful in ischemic lesions caused either by vasospasm or thrombosis (8). NG has also been shown to promote collateral circulation to the periphery of the lesion (8), as well as having analgesic properties (5). It is available in various formulations, such as patches, ointments or sprays, although the ointment formulation seems to allow for a safer profile of administration (18). In our institution, topical NG was initially available in the form of transdermal patches, which were applied off-label to ischemic lesions. Since mid-2022, however, NG patches have no longer been available. The hospital pharmacy is able to provide NG rectal ointment, acquired specifically for off-label use in ischemic lesions, although the process typically requires a few days. This circumstance accounts for the delays in treatment initiation observed in our study. The use of NG in pediatric ALI poses a challenge for clinicians, as there is no consensus nor are there guidelines defining optimal course of action. This has resulted in inconsistencies in the recommendations found in literature. For instance, while some authors suggest applying topical NG only to the demarcation line of ischemia (9), as a way to minimize side effects, others recommend its use across the entire lesion (19), to maximize treatment success. As the absorption of NG into the systemic circulation is thought to be proportional to the extension of the area of skin where the treatment is applied (8), it is reasonable to expect an increase in the occurrence of side effects when used on a larger surface area. However, it is important to note that newborns and children with skin conditions may exhibit variations in their absorption rate (4). In our study, NG was applied to the entire area of ischemia, and no adverse effects were reported in all seven patients.

Optimal dosage of NG has also been a subject of discussion among clinicians. It has been hypothesized that a dose of 0.12 mg/kg to 2.5 mg/kg of NG best enhances its vasodilation effects (20). Alternatively, other authors suggested the use of 4mm/kg or 1.22mg/kg (21). As most studies report the use of NG as an ointment not mentioning dosage, no conclusions on this matter can be made. Treatment duration recommendations are, again, variable across studies, ranging from mere hours to 29 days (4, 22). In our study, treatment was kept for as long as improvements in ischemia were observed, ranging between two to 20 days, which is consistent with other reports (18, 19, 20). Optimal time for treatment initiation remains ill-defined in literature (20); however, it is thought that NG should be initiated as soon as ALI is suspected, aiming to maximize treatment success (23). In fact, some studies suggest that irreversible damage may be sustained after only four to six hours of ischemia (24). Treatment delay in our study was up to four days in our population, which is consistent with other reports (18, 22). Delays occurred whenever the patient’s clinical status precluded treatment initiation or when NG wasn’t readily available in hospital pharmacy, as explained previously. Despite these delays, the overall prognosis was favorable, suggesting that NG may exert a beneficial effect even when initiated several days after the onset of ischemia.

Side effects of NG include methemoglobinemia and hypotension (13). These are more prominent within the first six hours following application, reflecting peak concentration (9, 25). A 2021 systematic review reported no methemoglobinemia cases in children treated with NG ointment (18). However, side effects were observed in children treated with NG patches (18). These differences may be attributed to the extended duration of the NG patch treatment. Premature newborns appear to have a higher risk for methemoglobinemia (25), as their skin exhibits increased permeability. However, it should be noted that the overall topical absorption of NG is minimal, thereby reducing the likelihood of adverse effects (7). In our study, no significant methemoglobinemia was found.

NG-related hypotension is a more common side effect, with some studies reporting up to 11% prevalence (7). The onset of these hemodynamic effects occurs at one hour after application and persists for a duration of up to six hours (26). None of our patients exhibited a decrease in blood pressure, making NG a possible safe treatment option in the treatment of ALI.

### Other treatment options

While several studies report on ALI treatment with NG alone, it is believed that other non-pharmacological and pharmacological interventions have a synergistic effect in the treatment of ALI (3, 17). Anticoagulation therapy has been used alongside topical NG as a way to improve reperfusion. While some authors suggest prescribing anticoagulation only in cases of occlusive thrombi (22), others argue that it may have beneficial effects in ALI (27). In our study, all children received full anticoagulation therapy, without bleeding complications. Close monitoring of aPTT or anti-Factor Xa may be helpful in dose adjustment, maximizing the anticoagulation potential while reducing bleeding risks. Thrombolytic therapy with tissue plasminogen activator is another pharmacological option (3), but some authors recommend its use only in progressive or unresponsive to anticoagulation ALI (22). Other authors express concern regarding bleeding risks, suggesting strict criteria for its use (6). Re-occlusion may be another potential problem with fibrinolytic therapy (6). Intravenous NG (18), prostaglandins (17) and hyperbaric oxygen (1) are other treatment interventions suggested in literature. Again, studies are scarce, and additional research is needed to establish optimal treatment. Nerve block performed by an experienced anesthesiologist may also be an alternative intervention for localized ischemia (17, 22), as this procedure induces vasodilation and inhibits vasoconstriction, and appears to have promising results. However, the success rate of this procedure has yet to be more accurately described, ranging from significant improvement of ischemic lesions (9, 23) to no effect and significant morbidity (22). Furthermore, only very experienced anesthesiologists may be able to perform this procedure, which may limit its accessibility. Vascular surgery is another therapeutic option, often discarded as it has been reported to have very poor outcomes in the neonatal population (3).

### Treatment success

Most studies report a complete or partial recovery when using NG in ALI treatment (4). A 2021 systematic review (18) found a 76% recovery rate in patients treated with NG ointment. Another systematic review from 2020 (22) reported a complete recovery in up to 83% of newborns treated for ALI. In our study, a complete recovery was achieved in four children (60%), which appears to be inferior to other reports. This discrepancy may be explained by the different inclusion criteria. While most studies report only on catheter-induced ALI, we included children with hypoperfusion induced ischemia as well. As different mechanisms are involved in the pathophysiology of ALI, it is reasonable to expect differences in treatment results as well. Another reason for the apparently lower success rate in our study may be patient age. Most studies report on ALI in newborns, who appear to have a higher skin permeability to nitroglycerin (22), and consequently presumably also a higher recovery rate. We included children of all ages, with only one newborn, and our eldest patient being 15 years old. Other various factors, such as extension of ischemia, comorbidities, treatment duration and the implementation of additional pharmacological and non-pharmacological measures may also have a role explaining these differences. Several studies have highlighted the importance of starting NG in the early stages of ischemia (7, 9), as treatment effects are limited after necrosis is established. In our study, time for treatment initiation suffered some delays as mentioned previously, which may be another explanation for differences in treatment success rate. Hospital pharmacies should consider stocking NG treatment options to minimize these delays. Notwithstanding, NG treatment proved to be a useful therapeutic intervention in our population, with the vast majority demonstrating at least partial recovery of ischemic lesions. This is consistent with literature reports (26). Some authors (1) have described a reperfusion-type injury in patients treated for ALI. This was not the case with our population. ALI in the pediatric population is associated with low amputation and mortality rates (10). If hemodynamic stability is assured, amputation should be delayed until necrosis is well established, as may sometimes be distal than originally appeared (1). Whenever this procedure is required, considerations about future prosthetics should be taken into account (1). In our study, amputation was performed in three children, two of them only partially. These children remain with residual scarring but without functional impairment. The third child underwent a lifesaving early amputation, as necrosis in both lower limbs had infected and presented significant hemodynamic consequences.

### Study limitations

This study presents several limitations. First, the small number of cases reflects the rarity of this condition and limits the generalizability of the findings. Second, it is plausible that distinct mechanisms of ALI result in heterogeneous outcomes and variable responses to treatment. In this report, ALI cases of all etiologies were analyzed collectively, which may have influenced the conclusions. Finally, as there are no international guidelines or consensus regarding management of ALI, all patients received the same therapeutic approach which may have further impacted the interpretation of our results. More studies are needed to clarify the best treatment approaches to these patients.

## Conclusion

Pediatric ALI is a rare condition sometimes seen in the ICU. Concrete data regarding incidence and optimal management are still scarce in literature, with some articles providing conflicting recommendations. Treatment success in ALI is thought to be multifactorial, with patient’s age, comorbidities, duration and extent of ischemia, and treatment used, all playing a role. Topical NG appears to be a beneficial and safe intervention in children with ALI of both vascular and nonvascular causes. Further studies are needed to strengthen the evidence base for the management of pediatric ALI.

### Key Points

- Pediatric acute limb ischemia (ALI) is a rare but serious complication in the intensive care setting, most often iatrogenic, following arterial catheterization.
- Topical nitroglycerin (NG) is a simple and safe therapeutic option that may promote limb reperfusion and limit ischemic damage.
- In this four-year experience, partial or complete recovery occurred in most cases treated with topical nitroglycerin, without adverse side effects.
- Early diagnosis, prompt removal of causative catheters, and combined use of anticoagulation and topical NG seem to optimize outcomes.
- Further multicentric studies are needed to define standardized treatment protocols for pediatric ALI management.

## References

[1] Arshad A, McCarthy MJ (2009). Management of limb ischaemia in the neonate and infant. Eur J Vasc Endovasc Surg.

[2] Friedman J, Fabre J, Netscher D, Jaksic T (1999). Treatment of acute neonatal vascular injuries – the utility of multiple interventions. J Pediatr Surg.

[3] Akingbola O, Singh D, Steiner R, Frieberg E, Petrescu M (2012). High-dose tissue plasminogen activator, topical nitroglycerin, and heparin for severe ischemic injury in a neonate. Clin Pediatr (Phila).

[4] Samiee-Zafarghandy S, van den Anker JN, Ben Fadel N (2014). Topical nitroglycerin in neonates with tissue injury: A case report and review of the literature. Paediatr Child Health.

[5] Varughese M, Koh TH (2001). Successful use of topical nitroglycerine in ischaemia associated with umbilical arterial line in a neonate. J Perinatol.

[6] Kayıran PG, Gürakan B, Kayıran SM (2013). Successful treatment of arterial thrombus in an extremely low-birth-weight preterm neonate. Pediatr Neonatol.

[7] Kim J, Lee JW, Kim DY (2020). Analysis of characteristics of peripheral arterial ischemia in premature babies and effects of nitroglycerin patch application. Child Health Nurs Res.

[8] Vasquez P, Burd A, Mehta R, Hiatt M, Hegyi T (2003). Resolution of peripheral artery catheter-induced ischemic injury following prolonged treatment with topical nitroglycerin ointment in a newborn: a case report. J Perinatol.

[9] Al Qurashi M, Al-Khotani A, Mohtisham F, AlRaddadi E, AlShaikh H, Hakami AY (2024). Digital Ischemia in an Extreme Preterm Infant Treated with Nitroglycerin Patch. Case Rep Pediatr.

[10] Lim S, Javorski MJ, Halandras PM, Kuo PC, Aulivola B, Crisostomo P (2018). Epidemiology, treatment, and outcomes of acute limb ischemia in the pediatric population. J Vasc Surg.

[11] Al Hinai M, Al Kindi I, Stephen E, Al Wahaibi K (2024). Pediatric Limb Ischemia: Our Experience from a Tertiary Hospital in Oman. Indian Journal of Vascular and Endovascular Surgery.

[12] Cerbu S, Bîrsăşteanu F, Heredea ER, Iacob D, Iacob ER, Stănciulescu MC (2018). Acute limb ischemia in neonates: etiology and morphological findings - short literature review. Rom J Morphol Embryol..

[13] Baserga MC, Puri A, Sola A (2002). The use of topical nitroglycerin ointment to treat peripheral tissue ischemia secondary to arterial line complications in neonates. J Perinatol.

[14] Maffei G, Rinaldi M, Rinaldi G (2006). Resolution of peripheral tissue ischemia secondary to arterial vasospasm following treatment with a topical nitroglycerin device in two newborns: case reports. J Perinat Med..

[15] Corbett M, Marshall D, Harden M, Oddie S, Phillips R, McGuire W (2019). Treating extravasation injuries in infants and young children: a scoping review and survey of UK NHS practice. BMC Pediatr.

[16] Weerasekera M, Lakmini C, Imbulana NS, Hettiarachchi KR, Sampath GU (2019). Topical nitroglycerine in management of peripheral ischaemia in a neonate following arterial cannulation. Sri Lanka Journal of Child Health.

[17] Shaniv D, Simpson-Lavy Y, Hershkovich Shporen C (2024). Management of iatrogenic acute limb ischaemia in the neonate. BMJ Case Rep.

[18] Sushko K, Litalien C, Ferruccio L, Gilpin A, Mazer-Amirshahi M, Chan AK (2021). Topical nitroglycerin ointment as salvage therapy for peripheral tissue ischemia in newborns: a systematic review. CMAJ Open.

[19] Kamar R, van Vonderen JJ, Lopriore E, Te Pas AB (2013). Nitroglycerin for severe ischaemic injury after peripheral arterial line in a pre- term infant. Acta Paediatr.

[20] Vivar Del Hoyo P, Sánchez Ruiz P, Ludeña Del Río M, López-Menchero Oliva JC, García Cabezas MÁ (2016). Nitroglicerina tópica en neonatos con lesiones isquémicas tras canalización de vasos [Use of topical nitroglycerin in newborns with ischaemic injuries after vascular cannulation]. An Pediatr (Barc).

[21] Wong AF, McCulloch LM, Sola A (1992). Treatment of peripheral tissue ischemia with topical nitroglycerin ointment in neonates. J Pediatr.

[22] Mosalli R, Elbaz M, Paes B (2013). Topical Nitroglycerine for Neonatal Arterial Associated Peripheral Ischemia following Cannulation: A Case Report and Comprehensive Literature Review. Case Rep Pediatr..

[23] Breschan C, Kraschl R, Jost R, Marhofer P, Likar R (2004). Axillary brachial plexus block for treatment of severe forearm ischemia after arterial cannulation in an extremely low birth-weight infant. Paediatr Anaesth.

[24] Sanches SMV, Aquino MA, Leite BL, Cerqueira MMBDF (2022). Conservative treatment of acute limb ischemia in a child – case report. J Vasc Bras.

[25] Mintoft A, Williams E, Harris C, Kennea N, Greenough A (2018). Methemoglobinemia during the Use of Glyceryl Trinitrate Patches in Neonates: Two Case Reports. AJP Rep.

[26] Alfraij A, Elseadawy M, Alghounaim M (2021). The effect of topical nitroglycerin on symmetrical peripheral gangrene in a pediatric patient. Clin Case Rep.

[27] Teo MC, Shah VA (2015). Digital ischaemia following inadvertent arterial cannulation of a peripherally inserted central catheter in a very low birth weight infant. Singapore Med J.

